# Banknote authenticity is signalled by rapid neural responses

**DOI:** 10.1038/s41598-022-05972-8

**Published:** 2022-02-08

**Authors:** Daniel B. Dodgson, Jane E. Raymond

**Affiliations:** School of Psychology, University of Birmingham, Edgbaston, Birmingham, B15 2TT UK

**Keywords:** Decision, Perception

## Abstract

Authenticating valuable objects is widely assumed to involve protracted scrutiny for detection of reproduction flaws. Yet, accurate authentication of banknotes is possible within one second of viewing, suggesting that rapid neural processes may underpin counterfeit detection. To investigate, we measured event-related brain potentials (ERPs) in response to briefly viewed genuine or forensically recovered counterfeit banknotes presented in a visual oddball counterfeit detection task. Three ERP components, P1, P3, and extended P3, were assessed for each combination of banknote type (genuine, counterfeit) and overt response (“real”, “fake”). P1 amplitude was greater for oddballs, demonstrating that the initial feedforward sweep of visual processing yields the essential information for differentiating genuine from counterfeit. A similar oddball effect was found for P3. The magnitude of this P3 effect was positively correlated with behavioural counterfeit sensitivity, although the corresponding correlation for P1 was not. For the extended P3, amplitude was greatest for correctly detected counterfeits and similarly small for missed counterfeits, incorrectly and correctly categorised genuine banknotes. These results show that authentication of complex stimuli involves a cascade of neural processes that unfolds in under a second, beginning with a very rapid sensory analysis, followed by a later decision stage requiring higher level processing.

Counterfeit refers to objects that closely resemble well-recognised, valued objects but are nevertheless different in subtle ways. Not only is the discrimination of counterfeit objects from their genuine counterparts critical for food (plant or prey) and predator identification for many animal species^1^, it is increasing important for humans due to the rapid increase of counterfeit goods in contemporary marketplaces^2,3^ and use of faked images on social media^4^. In these human scenarios, counterfeit are unauthorised reproductions of recognised products that have been manufactured with the specific intention to deceive others. Discriminating counterfeit from genuine objects is a demanding perceptual and cognitive task that pits rapid, low-level sensory analysis against slower, high-level object recognition mechanisms. On the one hand, cues to counterfeit (‘tells’) are typically subtle variations of one or more sensory features, e.g., altered hue or distortion of an object part. Yet, the brain’s high-level object recognition mechanisms routinely discount many such subtle sensory variations as they are usually irrelevant to object classification^5^. For example, a chair or even a particular chair is easily recognised when viewed from different viewpoints or under different sources of illumination that may subtly change the chair’s hue. Even though the retinal image of the chair may be substantially different under different viewing conditions, its recognition remains robust, a phenomenon known as object constancy (for a review see^6^). Indeed, high-level object recognition is thought to involve accumulating just enough sensory information^7^ to allow a match between incoming information and stored internally represented object protypes or exemplar sets^8^. To gain efficiency, this matching mechanism depends on selecting only highly relevant sensory information that is diagnostic of object category, ignoring sensory variations that reflect situational factors, such as lighting, object orientation, observer viewpoint, or the presence of occluding objects or shadows^9^. Although this process allows rapid, flexible object recognition, it impedes counterfeit detection as the latter depends largely on detection of subtle sensory cues.

High-level object recognition is widely assumed to involve predictive coding^10,11^. In this view, the brain anticipates the appearance of an object based on learned and contextual factors and sets information processing mechanisms to selectively boost sensitivity to relevant sensory cues and reduce sensitivity to non-relevant cues. This may be achieved by a series of feedback loops whereby high-level cortical networks exert object-specific top-down modulatory effects that act on initial, incoming sensory signals about 100 ms after stimulus onset^12^. Such processes depend on experience and indeed, expertise in recognizing specific types of complex visual objects is associated with the appearance of early ERP components that are not found in novices^13^. Although the incoming ‘forward sweep’ of sensory information may be different for counterfeit versus genuine objects and could thus provide a useful signal to the brain, differences could be obscured subsequently if top-down object-oriented filtering were imposed. However, if incoming signals were sufficiently unexpected, they could alert high-level strategic mechanisms to alter or widen object recognition processes^9^, prolonging the high-level analysis needed for object categorisation and thereby facilitating counterfeit detection. This notion of a two-stage process in counterfeit detection that begins with sensory cue detection, followed by greater cognitive engagement of object analysis mechanisms has been previously proposed to account for behaviour^14^ and eye movements^15^ made during a banknote authentication task. However, direct evidence of the underlying neural mechanisms that could mediate such a two-stage process is lacking.

To probe the underlying neural processes involved in object authentication, we measured how sensory signals that differentiate counterfeit from genuine are propagated through the brain using electrophysiology combined with a banknote authentication task. We studied banknote authentication as banknotes provide complex, yet familiar stimuli and the task has practical everyday implications. We recorded event related potentials (ERPs) with scalp electrodes and presented stimuli using an oddball paradigm, a procedure widely used to study stimulus categorisation^16–18^. We chose this paradigm because it mimics the real-world conditions wherein encountering a counterfeit is rare and encountering genuine notes is frequent. In this procedure ERPs produced in response to standard (frequent) stimuli are compared to those produced in response to oddball (infrequent) stimuli when presented in an intermixed, non-predictable series. Here, genuine banknotes served as standards and convincing, forensically recovered counterfeits served as oddballs. Participants judged each note as real or fake on each presentation.

Specifically, we focussed on three ERP components: the P1, the P3, and the extended P3 (sometimes referred to as the “prolonged P3” or “slow wave”)^6^. P1, the first positive-going wave, is observed over occipital electrodes about 100 ms post-stimulus onset and is driven largely by the physical characteristics of stimuli, thus reflecting the forward sweep^19^. We predicted differences in this component for genuine (standard) versus counterfeit (oddball) banknotes, regardless of the participant’s eventual overt authenticity judgement. The second ERP component, P3, is the third positive-going ERP peak typically observed around 300–650 ms post stimulus onset and is maximal at central and parietal electrode sites. Unlike P1, P3 is influenced endogenously, being greater for unexpected (oddball) than expected (standard) stimuli^16,20^, and is presumed to occur during or after stimulus categorisation and probably before response selection^17^. The P3 is also sensitive to the difficulty of the target to nontarget discrimination^18^, exhibiting longer latencies for more difficult stimulus categorisations^21^. Here, we predicted P3 oddball effects to be evident and to be correlated with the accuracy of overt authenticity judgements.

Previous studies show that when stimuli are especially difficult to discriminate, as in our study, P3 oddball effects are prolonged beyond the typical P3 time window^6,22,23^. An extended P3 has been interpreted as evidence of the onset of a second level of cognitive engagement that commences after the initial feature matching categorisation thought to invoke the primary P3^6,24–26^. We therefore predicted the oddball effect to be observable during the extended P3 time interval, especially for correctly detected counterfeit. P3 and the extended P3 have been closely linked to conscious stimulus categorisation suggesting that these components could depend on overt authentication decisions^27^. To investigate, we analysed these components separately for trials resulting in each possible response (“real”, “fake”) for each note type (genuine, counterfeit). “Fake” responses to genuine notes reflect either guesses or decisions made without sensory counterfeit tell signals; “real” responses to counterfeits could also reflect guesses but they may also indicate that an available sensory tell signal was overridden by a later categorisation process. We predicted that only when counterfeits were correctly and consciously reported would prolonged P3 effects be observed, as these are thought to reflect sustained attention and working memory updating used for complex processing^25,26^. Such high-level, conscious processing of *bone fide* counterfeits (i.e., supported by sensory evidence) would be highly task relevant and beneficial, and therefore should be associated with a large extended P3.

In addition to the univariate ERP analysis, multivariate pattern analysis (MVPA) can be used to decode differences in the mental representations^28^ associated with genuine and counterfeit banknotes, and between “real” and “fake” responses to authentic and counterfeit banknote across all EEG channels. Here, MVPA was used to corroborate the ERP approach and to investigate the time course of judgements for counterfeit and genuine banknotes. Specifically, MVPA was used to assess temporal generalizations that indicate the stability of genuine and counterfeit representations across time^29^. We predicted that the time window for accurate decoding of genuine and counterfeit banknotes would mirror that of the P3 component of the ERP because of the high-level processing required to differentiate the stimuli. We further predicted that stable representations, and therefore accurate decoding of responses to counterfeit banknotes would be protracted compared to the response to genuine banknotes. This is expected because identification of counterfeit flaws should involve secondary processing, sustained attention, and working memory. These predicted patterns of univariate and multivariate electrophysiological response would not only provide insight into authentication processes in a wide range of contexts but also inform object categorisation processes more generally.

## Results

Twenty-three British adults were presented with a series of brief (300 ms) UK banknotes image presentations (576 of genuine and 144 of counterfeit; half being £20; remaining, £50). Note type (genuine, counterfeit) was fully crossed with denomination; notes for each denomination were presented in separate blocks. Each note was presented upright and front facing in a short movie clip. During each clip, the note rotated slightly around its y-axis, emphasising the visual characteristics of the primary security features. The principal measures were overt counterfeit sensitivity (d′) and ERP components; each was analysed for effects of denomination and note type.

### Behavioural data

As viewing time was brief*,* counterfeit sensitivity was relatively low (mean £20 d′ = 0.95 (s.d. = 0.83), mean £50 d′ = 0.94 (s.d. = 0.97), and similar for each banknote denomination (*t*(1, 22) = 0.001, *p* = .981). Response times (RT) were slower for counterfeit (mean = 905 ms, s.d. = 206) than for genuine notes (mean = 823, s.d. = 206; *t*(22) = 2.739, *p* = .012), an effect that may have been partly due to use of the left hand for "fake” responses, but did not vary significantly with denomination (*F*(1,22) = 2.315, *p* = .142, ηp^2^ = 0.095). RT effects of banknote type did not interact significantly with denomination (*F*(2, 44) = 2.024, *p* = .144, ηp^2^ = 0.084).

### ERP data

#### P1 component at electrode location Oz

**Figure 1:**
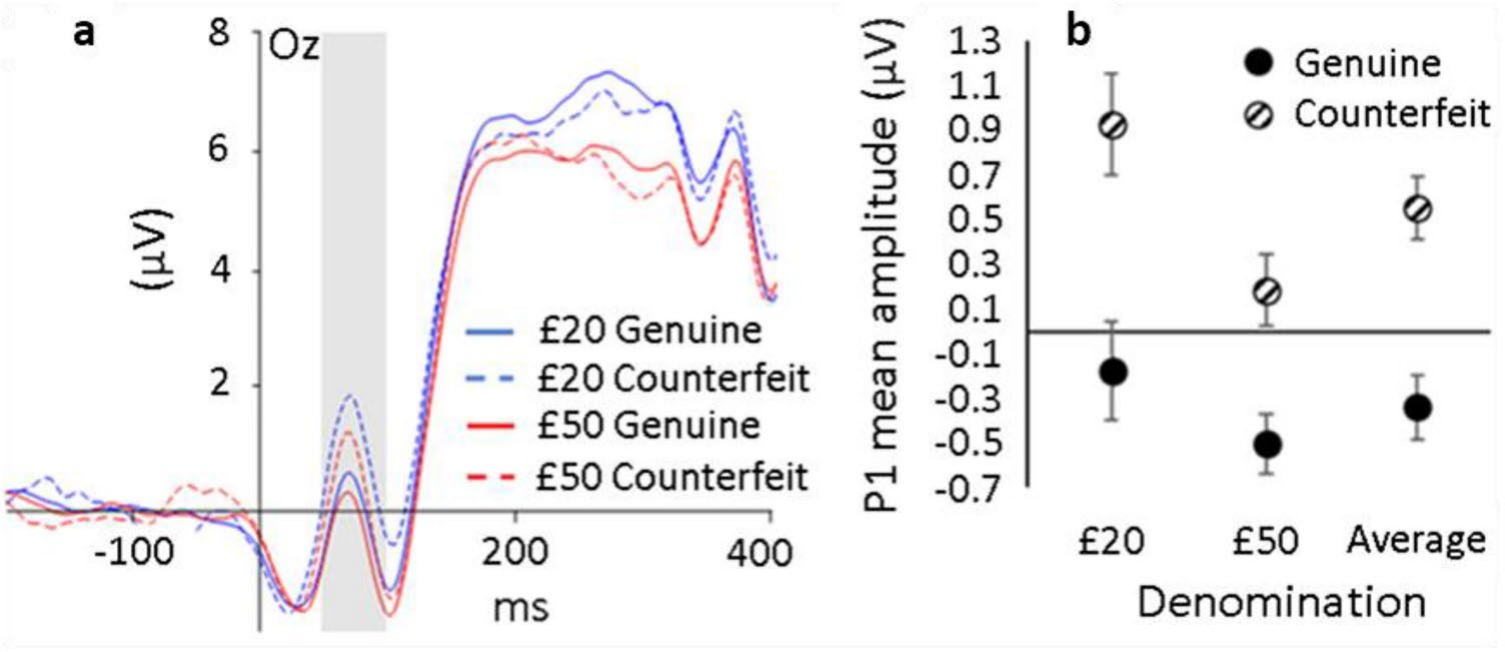
(**a**) Group mean amplitude (µV) of the ERP for each denomination and authenticity at electrode location Oz (where P1 was measured) plotted as a function of time relative to banknote presentation onset (0 ms). Blue and red lines represent £20 and £50 note conditions, respectively. Solid and dashed lines represent genuine and counterfeit banknote conditions, respectively. Positive is plotted up. The shaded rectangle indicates the time interval for P1. (**b**) Group mean amplitude at the P1 component plotted for each authenticity and denomination (including the average of each denomination). Error bars represent ± 1 within-subject S.E of the mean^30^.

ERPs obtained for each note condition are shown in Fig. 1a. For both the £20 and £50 notes, mean amplitude of the P1 component of the ERP was significantly larger for counterfeit (dashed lines; £20 = 0.924 µV, £50 = 0.183 µV) versus genuine (solid lines; £20 = − 0.177 µV, £50 = − 0.502 µV) notes (*F*(1, 22) = 10.085, *p* = .004, ηp^2^ = 0.314), showing a clear oddball effect. Across authenticities, P1 mean amplitude was also significantly larger for £20 (0.373 µV) compared to £50 (0.339 µV) notes (*F*(1, 22) = 6.389, *p* = .019, ηp^2^ = 0.225) as shown in Fig. 1b. The magnitude of the P1 difference between counterfeit and genuine notes (oddball effect) was not significantly different for £20 and £50 notes (*F*(1, 22) = 2.116, *p* = .160, ηp^2^ = 0.088). The correlation between P1 mean difference scores and d′ was low and non-significant (*r* = 0.249, n = 23, *p* = .251).

#### P3 component at electrode location Pz

**Figure 2:**
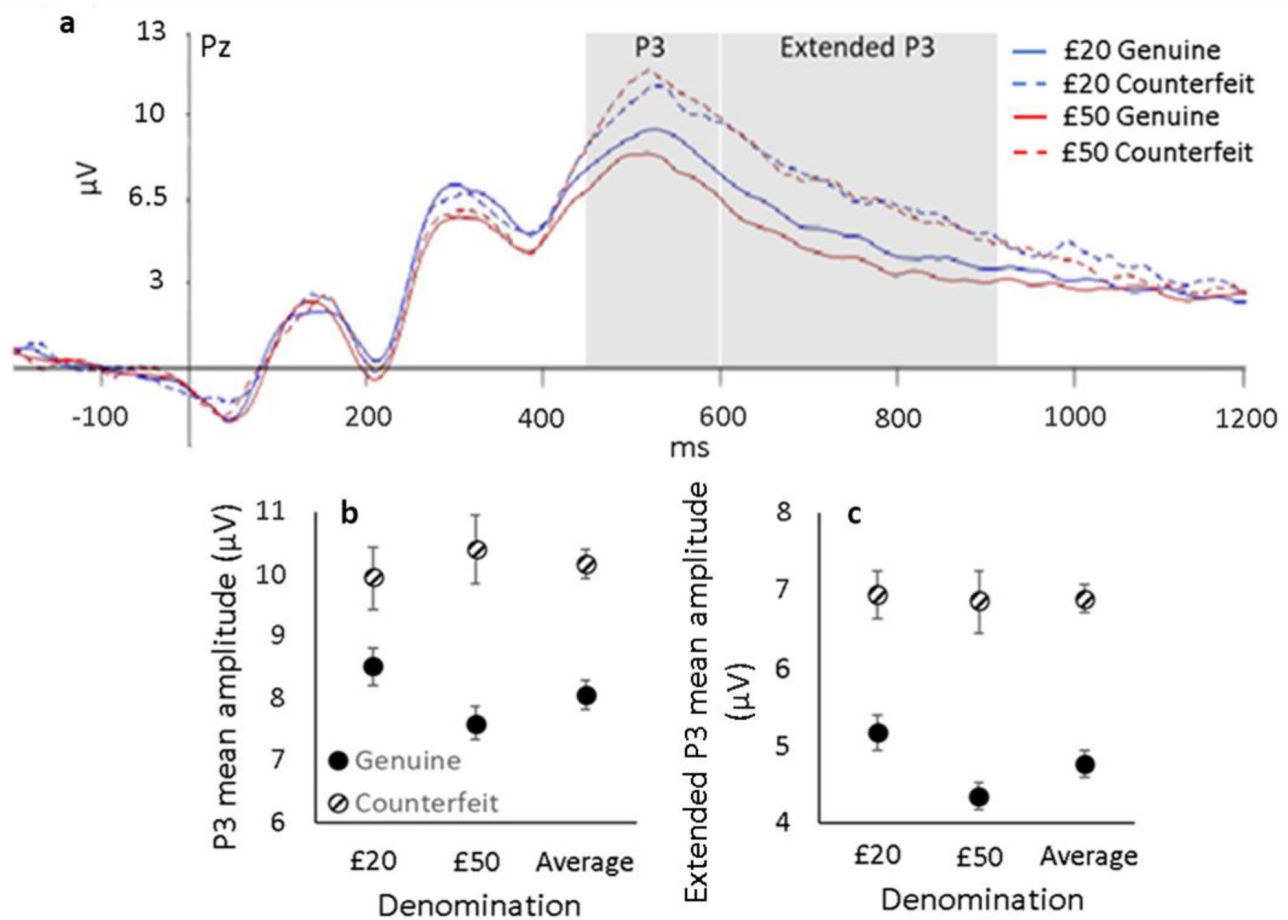
(**a**) ERP for each denomination and authenticity at electrode location Pz, where the P3 component mean amplitude within the 450–600 ms window is calculated. Blue lines represent £20 notes, red lines represent £50 notes. Solid lines are genuine notes, dashed lines are counterfeit notes. Positive is plotted up. Shaded rectangle indicates the general time interval for each component. Group mean amplitude at the P3 (**b**) and the extended P3 (**c**) components plotted for each authenticity and denomination (including the average of each denomination). Error bars represent ± 1 within-subject S.E of the mean^30^.

See Fig. 2a. For both the £20 and £50 notes, the P3 component of the ERP was significantly larger for counterfeit (£20 = 9.93 µV, £50 = 10.39 µV) versus genuine (£20 = 8.51 µV, £50 = 7.61 µV) notes (*F*(1, 22) = 19.543, *p* < .001, ηp^2^ = 0.470; £20: *t*(22) = 2.337, *p* = .029, £50: *t*(22) = 4.224, *p* < .001) as shown in Fig. 2b. There was no difference in the magnitude of the P3 oddball effect (counterfeit v. genuine) for £20 and £50 notes (*t*(22) = 1.622, *p* = .119).

**Figure 3:**
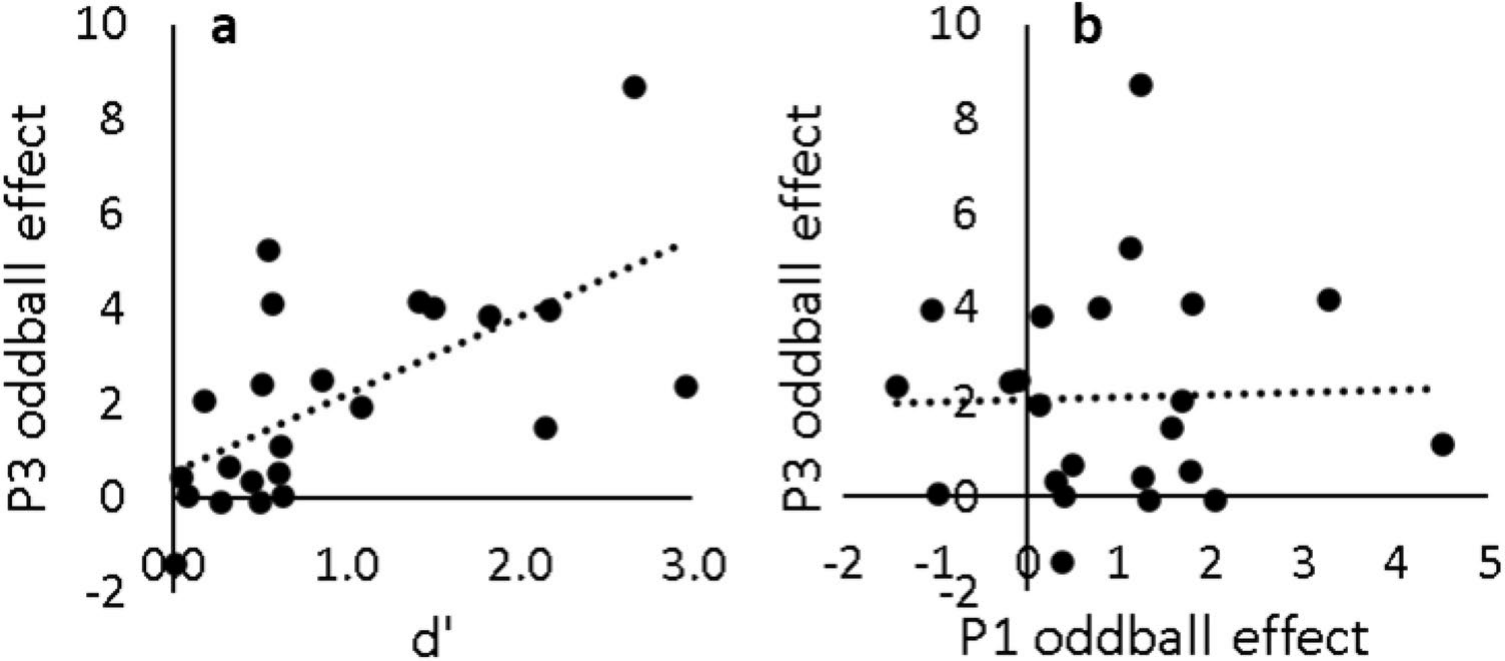
(**a**) Mean counterfeit sensitivity (d′) collapsed across all denominations and counterfeit qualities as a function of the P3 oddball effect (counterfeit—genuine) at Pz. (**b**) P3 (450–600 ms) oddball effect at electrode Pz as a function of P1 (50–100 ms) oddball effect at electrode Oz. Each dot represents data from one participant.

The P3 oddball effect correlated strongly with d′ (*r* = 0.629, *p* = .001) as shown in Fig. 3a. Figure 3b shows that there was no correlation between the oddball effects observed in the P1 and P3 components.

#### Extended P3 component at electrode location Pz

As can be seen in Fig. 2a, the difference in the waveforms for genuine and counterfeit notes was not limited to the traditional P3 time window (450–600 ms). Here, we observed large differences in the ERPs associated with genuine versus counterfeit notes until around 900 ms after the onset of the banknote’s presentation. Indeed, the mean amplitude of the extended P3 was significantly (*t*(22) = 6.133, *p* < .001) higher for counterfeit (mean = 6.89 µV) versus genuine (mean = 4.76 µV) notes as shown in Fig. 2c. Interestingly, this extended P3 effect did not correlate with behavioural counterfeit sensitivity (d′, r = 0.255, n = 23, *p* = .240), suggesting that this signal reflects the believed authenticity rather than the actual authenticity of the notes.

#### Response-based analysis

**Figure 4:**
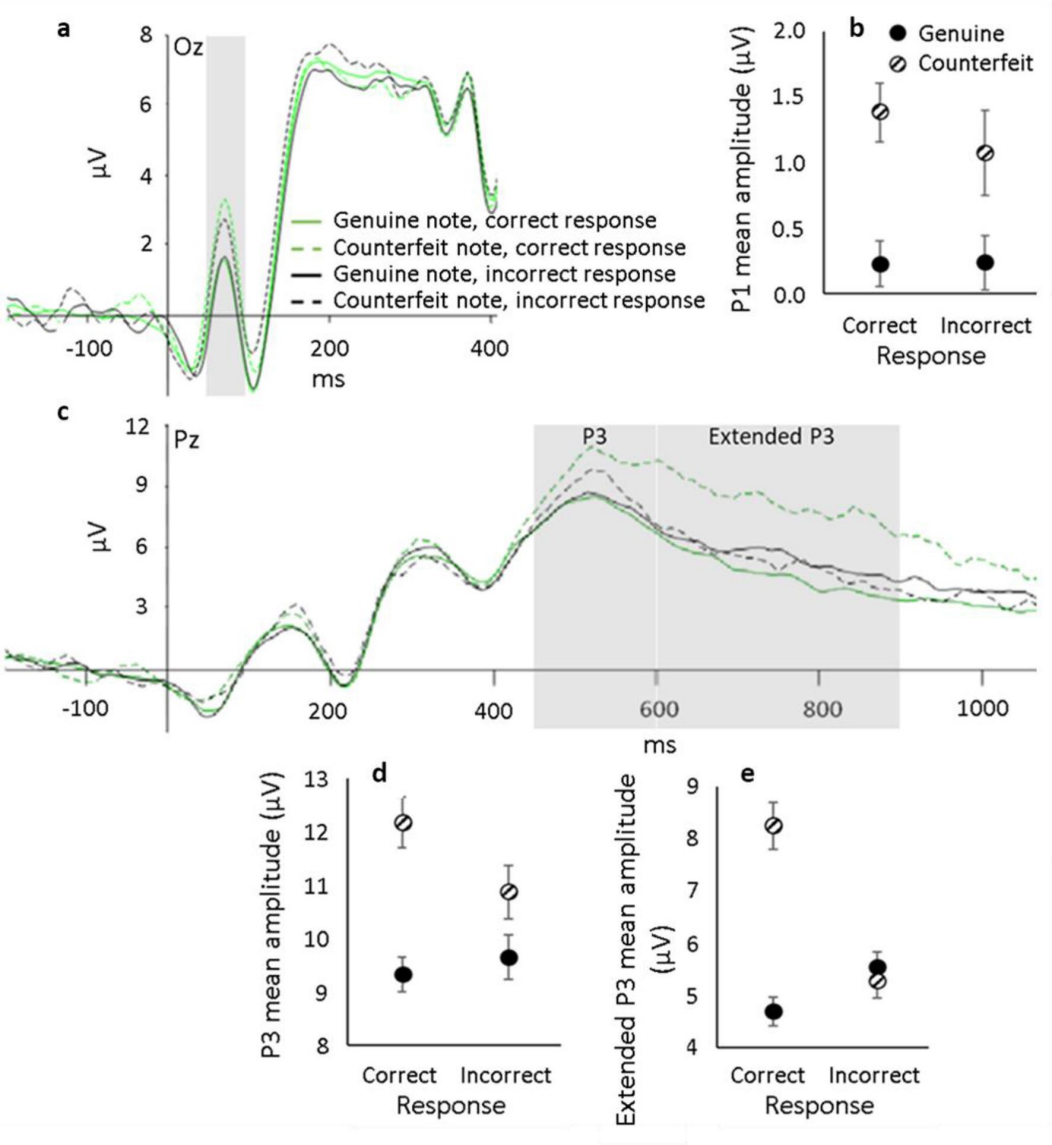
ERPs measured at two different electrode sites for each genuine (solid lines) and counterfeit (dashed lines) notes when the response was correct (green) and incorrect (black). Positive is plotted up. Shaded rectangle indicates the general time interval for each component. (**a**) ERPs obtained from electrode site Oz where effects on the P1 were observed. (**b**) Group mean amplitude at the P1 component plotted for each authenticity and the participants’ response. (**c**) Potentials obtained from electrode site Pz where effects on the P3 and extended P3 components (around 500 ms) were observed. Group mean amplitude at the P3 (**d**) and the extended P3 (**e**) components plotted for each authenticity and the participants’ response. Error bars represent ± 1 within-subject S.E of the mean^30^.

*P1 and P3 components.* If brain states drive behaviour, then ERPs might be expected to differ based on overt behavioural response rather than on the sensory information available. To investigate, ERPs at Oz and Pz were computed separately depending on response (“real”, “fake”) for each physical note condition (genuine, counterfeit), collapsed across denominations. See Fig. 4a.

Analyses showed that the P1 component (Fig. 4a) was unaffected by participant’s response (*F*(1, 18) = 0.318, *p* = .580, ηp^2^ = 0.017) and there was no interaction between authenticity x response (*F*(1, 18) = 0.503, *p* = .487, ηp^2^ = 0.027) (Fig. 4b). This finding suggest that this very early component may underpin the initial accumulation of evidence needed to evoke a sense of suspicion towards potentially counterfeit notes. A similar picture holds for the P3 component (Fig. 4c, d). It was unaffected by response category (*F*(1,18) = 1.639, *p* = .217, ηp^2^ = 0.083), and showed a non-significant interaction between note authenticity and response (*F*(1, 18) = 1.764, *p* = .201, ηp^2^ = 0.089).

Importantly, differences between the genuine and counterfeit note conditions for P1 and P3 components remained significant even when analyses were confined to trials resulting in a “real” response. Specifically, when ERPs generated in response to genuine notes that resulted in “real” (correct) responses were compared to ERPs generated in response to counterfeit notes that also resulted in a “real” (incorrect) response, both the P1 (*t*(18) = 3.101, *p* = .006) and P3 (*t*(18) = 2.695, *p* = .015) were significantly different. This indicates that from approximately 50–500 ms after onset of the banknotes image, neural information accurately coding physical authenticity of the banknote was available in the brain but failed to influence the overt behavioural decision appropriately.

#### Response-based analysis: extended P3 component

See Fig. 4c. In marked contrast to the effects found for P1 and P3 components, analysis of extended P3 amplitudes showed that ERPs generated in response to counterfeit notes and accompanied by a correct “fake” response were markedly greater (*F*(1, 18) = 69.562, *p* < .001, ηp^2^ = 0.487) compared to all other note-response combinations (all *t*s > 4.531, all *p*s < 0.001) as shown in Fig. 4e. This extended P3 component likely reflects conscious detection of the oddball (counterfeit) after many sources of information (e.g., expectation) have been combined. However, it is unlikely to reflect the decision outcome itself because counterfeit judged to be fake and genuine banknotes also judged to be fake (error) produced different extended P3 amplitudes. (Compare the green dotted line with black solid line in Fig. 4c.).

### Classification accuracy data

#### Genuine versus counterfeit banknotes

**Figure 5:**
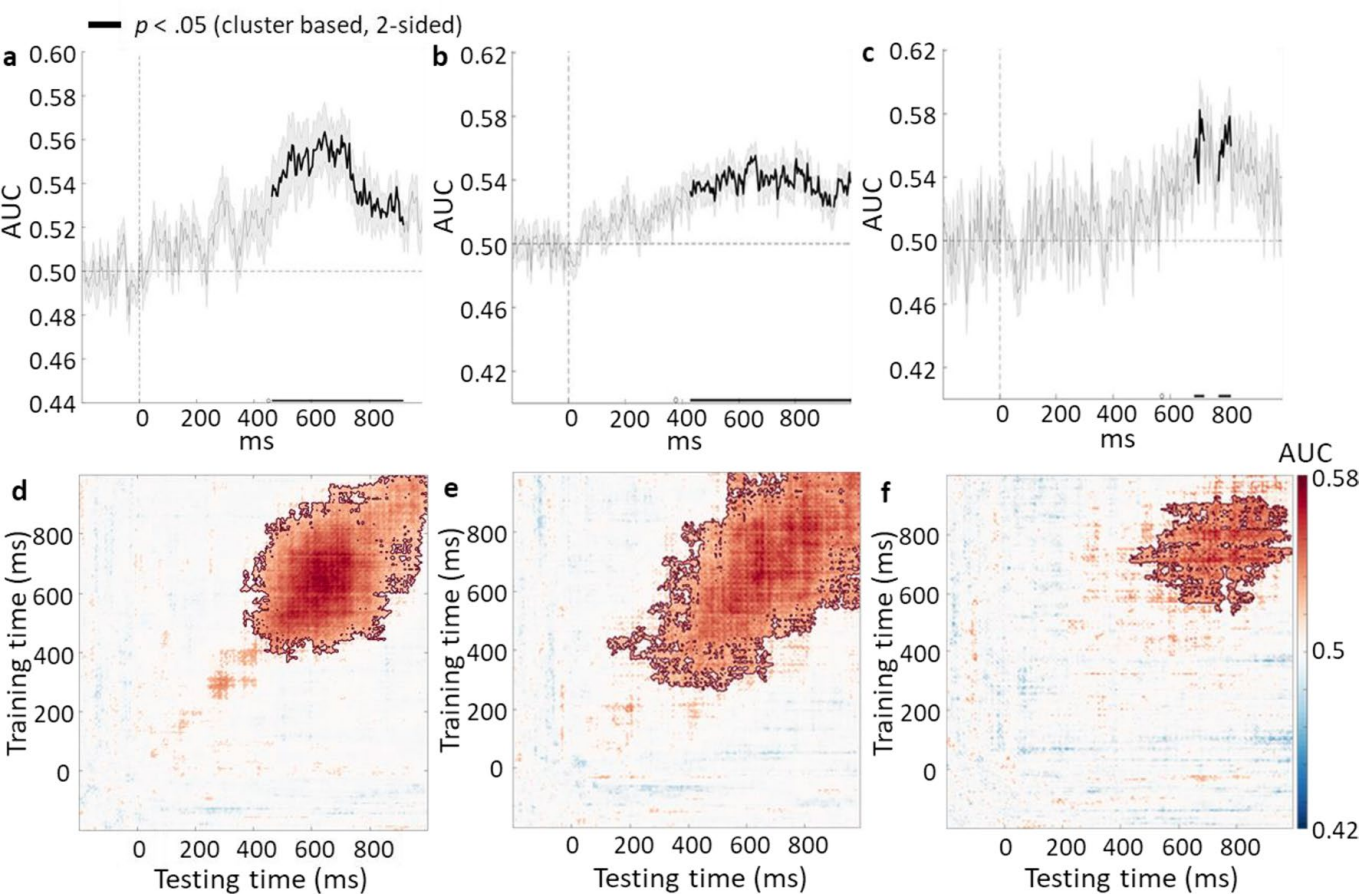
The top panels show the classification accuracy across time and the bottom panels show the temporal generalization matrices between (**a** and **d**) genuine and counterfeit banknotes; (**b** and **e**) “real” and “fake” responses to genuine banknotes; and (**c** and **f**) “real” and “fake” responses to counterfeit banknotes.

See Fig. 5a and d. The authenticity of the banknote could be predicted significantly (*p* < .05) above chance as a stable representation between 450 and 900 ms following the presentation of the banknote, an interval that corresponds to the timing of the P3 component of the ERP and likely reflects the dominance of this component across the scalp in the EEG.

#### Response-based analysis: genuine and counterfeit banknotes

When the banknote was genuine (Fig. 5b, e), the response (“real” or “fake”) could be predicted significantly better than chance between 380 and 1000 ms following the presentation of the banknote. However, when the banknote was counterfeit (Fig. 5c, f), the response could not be predicted significantly better than chance until considerably later than for genuine banknotes. For counterfeit banknotes, predictions of the response were significantly better than chance from 680 to 800 ms following the onset of the banknote, a finding that suggests decisions about the authenticity of counterfeit banknotes take longer than that for genuine banknotes.

## Discussion

Authentication involves a combination of sensory discrimination and higher-level object categorisation to determine if a valued object is genuine or counterfeit. Here, for the first time, we monitored human electrophysiological responses as people authenticated briefly presented genuine banknotes and forensically recovered counterfeit. Banknotes were presented in a visual oddball paradigm involving genuine banknotes as standards and counterfeit as oddballs. ERPs, specifically P1, P3, and the extended P3, to standard stimuli (genuine) or oddballs (counterfeit) were assessed and compared so that the neural signature differentiating genuine from counterfeit, i.e., the authentication signal, could be tracked temporally after stimulus onset. We then linked the magnitude of the authentication signal with performance to determine the utility of its information for the behavioural task. Lastly, we compared ERPs for each note (genuine, counterfeit) and response type (“fake”, “real”) to examine whether variations in component amplitude reflected stimulus type, overt response, or a combination. Results reveal that a cascading series of neural signals unfolds during authentication of a counterfeit note. Very quickly after viewing the banknote, sensory information that differentiates counterfeit from genuine is coded and then top-down neural processes, e.g., expectation, appear to modulate how this early sensory signal contributes to overt authentication judgements. Lastly, authentication decisions for genuine banknotes are made more quickly than those regarding counterfeit banknotes. When the latter are identified, protracted high-level engagement appears to be evident, a process that may support category learning.

To reduce the likelihood that effects found here were limited to a particular banknote design or its specific counterfeit, two different banknotes (UK £20 and UK £50) were examined, creating a replication within the study. No differences due to denomination were uncovered even though the two notes and their counterfeits differed in colour, size, and authentication-relevant design features.

The authentication task used here was difficult, attention-demanding, and performance averaged only 65% correct, (with a group average d′ = 0.94, s.d. = 0.86). Nevertheless, P1 responses provide clear evidence that the sensory information needed to differentiate counterfeit from genuine was available to the brain within 100 ms after stimulus presentation. P1 oddball responses were significantly greater than P1 standard responses, an effect that is widely considered to reflect purely sensory processes with little or no top-down or attentional influence^19^. Our novel finding of a P1 oddball effect in the context of authentication shows that the brain can rapidly and automatically register subtle sensory differences between genuine and counterfeit, a finding consistent with other observations that P1 amplitude is extremely sensitive to the physical characteristics of stimuli^31^. Interestingly, these early pre-conscious brain signals are insufficient to support overt authentication behaviour as the magnitude of an individual’s P1 effects did not correlate with their counterfeit sensitivity performance (d′). However, as these early signals reflect a mismatch between the expected standard and current sensory data of a counterfeit, they may serve to trigger or direct high-level, selective attention to specific scene elements so that appropriate overt behaviour response, e.g., banknote rejection, is more likely to occur.

Evidence to support this notion was found in the oddball effects for P3. P3 amplitude is widely viewed as an index of post-sensory processes, including stimulus categorisation^25^, working memory^32^, conscious perception^33^, and conscious recognition of the decision category^27,34^. As with P1, P3 responses measured here were greater for counterfeit versus genuine banknotes indicating that the P3 oddball effect also indexes available sensory differences. Here, the P3 was observed relatively late, between 450 and 600 ms post-stimulus onset, probably reflecting the difficulty of the discrimination task^25^ and possibly the modest temporal variability in the availability of cues in the slowly rotating banknotes. When compared to the P1 oddball response, however, the P3 authentication signal appears to code information with critical task relevance. This probably reflects active selective attention of specific information within the banknote. The magnitude of the P3 oddball effect correlates strongly with counterfeit sensitivity (d′) whereas its P1 counterpart does not. In other words, finding that the P3 oddball effect is predictive of performance is consistent with the conjecture that top-down attention processes influence neural activity during the interval between 450 and 600 ms post-stimulus, but not earlier. Additional support for this view is that MVPA classification of the banknote authenticity was not accurate until the P3 time window. The lack of a stable mental representation prior to the P3 time window suggests that without support from top-down processes, bottom-up signals are insufficient to decipher the authenticity of the banknotes. Furthermore, the P1 oddball effect did not correlate with the P3 oddball effect.

Although the P3 data reported here are consistent with views that P3 indexes post-sensory, conscious category recognition, data obtained thereafter (extended P3; 600–900 ms) appear to reflect an elaboration of categorical processing and working memory. Indeed, further processing after P3 is generally associated with tasks requiring complex categorical decisions^6^, as required here. Although P1 and P3 magnitude for each stimulus type (genuine, counterfeit) did not differ depending on response (“fake”, “real”), extended P3 amplitude was determined by an interaction of stimulus type and response. Specifically, correctly detected counterfeit produced a greater extended P3 than all other combinations. This means that a large extended P3 did not merely reflect a decision to report a banknote as fake, as it was larger when counterfeit was classed as ‘fake’ than when genuine notes were classed as ‘fake’. Moreover, it also cannot reflect a purely stimulus driven response, as it was greater for correct than incorrect counterfeit trials, even though the stimuli were similar in both cases. The pattern of extended P3 effects found here suggests that this component indexes confident counterfeit decisions that had been supported by sensory authentication signals extracted earlier in processing, rather than counterfeit judgements based on guessing or expectation.

The extended P3 has previously been attributed to the instigation of a secondary cognitive tasks that occur after initial stimulus categorisation^24^. In the present context extended P3 effects may reflect additional, confirmatory, internal elaboration of counterfeit stimulus representations using higher-order selection mechanisms when differences between the sensory input and the expected mental template of a genuine note are present. In this view the extended P3 could reflect serial searching of the memory representation of the note for information that confirms the counterfeit judgement. This would be consistent with eye-movement patterns found during banknote authentication that suggest confirmatory visual scanning^15^ and with extant views of serial visual search strategies^35,36^. The later latency of the MVPA classification accuracy of the behavioural response when the banknote was counterfeit compared to genuine also suggests that genuine banknotes can be classified quickly, whereas counterfeit banknotes require protracted processing, likely involving higher-order processes such as selective attention, working memory, and long-term memory. Another possibility is that the extended P3 reflects an initial stage in a learning process whereby cues to counterfeit are selectively maintained in working memory so that long term memory representations for supporting accurate authentication in the future can be established^37^. These ideas concerning the functional significance of the extended P3 in this experiment are complementary and it is likely that this component reflects a combination of both processes.

Collectively, the ERP findings support a behavioural model of banknote authentication, called the Suspicion Initiated Model of Banknote Authentication (SIMBA^14^). In this view, rapidly processed sensory data are matched to an internal template of an expected genuine exemplar. When discrepancies are present, signals (‘suspicions’) are used to activate greater cognitive engagement and visual search behaviour so that more information can be accumulated. When early sensory evidence of counterfeit is lacking, the expectation that the note is genuine is confirmed and the note is not further analysed. This model has important implications for consumer-directed anti-counterfeit measures used in banknote designs. Specifically, security features, i.e., hard to duplicate banknote features e.g., holograms, are used to provide an obvious indication of authenticity. Nearly all security features currently in use on banknotes require strategic scrutiny of fine details to confirm authentication. They thus require high-level cognitive engagement that the model suggests would be unlikely if other rapidly available cues on the banknote did not raise counterfeit suspicion immediately after viewing onset. The data reported here support this authentication model by revealing a cascade of processing stages. Specifically, the P1 oddball effect shows evidence that the initial feedforward sweep of information processing can differentiate genuine and counterfeit notes. However, this information alone is insufficient to support a counterfeit identification. Subsequent processing involving top-down information to guide selection and to elaborate the representation of a counterfeit is found in the P3 and especially the extended P3 response. The pattern of data reported here are thus consistent with SIMBA and have direct implications for anti-counterfeit measures related to the design of banknotes and other high value items.

## Method

### Participants

26 students from the University of Birmingham (9 males; mean age = 19.0 years [SD = 0.9 years, range = 18–22 years]) took part in exchange for £16 or course credit after providing informed consent. All procedures were approved by the University of Birmingham Research Ethics Committee (ERN_17-1673) and were performed in accordance with their guidelines. Data from three participants were excluded from all analyses; two performed below chance (d′ < 0), indicating failure to follow instructions, and one withdrew early, leaving 23 complete data sets.

### Apparatus and materials

A *DELL* XPS-15 laptop PC (screen resolution: 3840 × 2160; refresh rate: 60 Hz) using custom software created in Matlab (*The MathWorks, Inc*) and Psychophysics toolbox^38^ determined stimulus presentation order, recorded data, and presented stimuli. Authentication responses were recorded using an external standard QWERTY keyboard.

**Figure 6:**
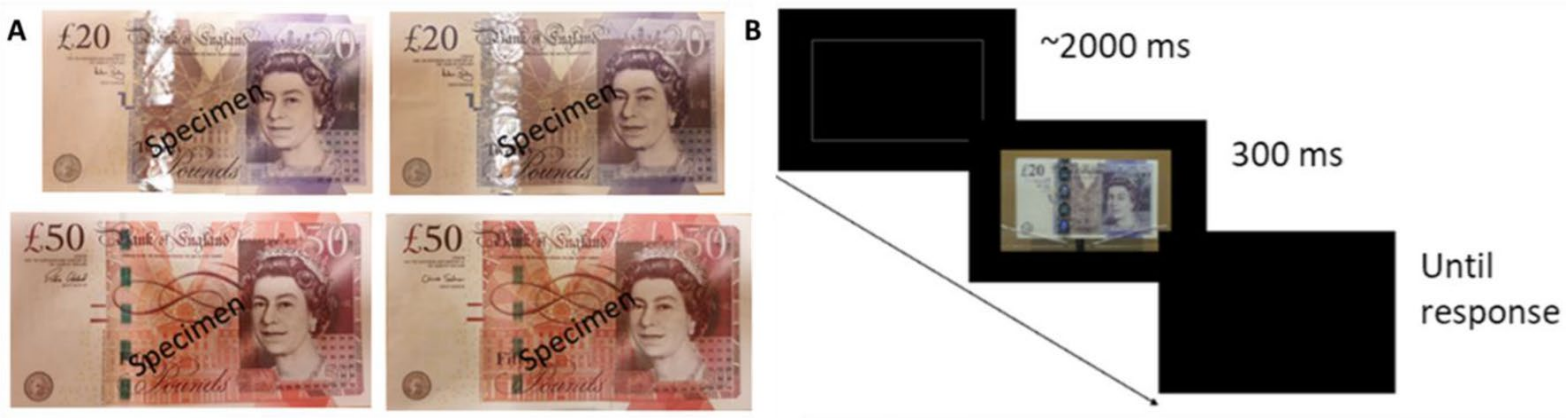
(**A**) Example Images of the genuine (left) and counterfeit (right) GBP £20 (top) and £50 (bottom) notes. (**B**) Trial sequence in the Authentication Task. The trial image of the £20 note is a still from a video clip showing a genuine note. The word “specimen” was never present during the task.

All banknotes were presented as digital video images. 48 different genuine banknotes (24 x £20 and 24 x £50 notes) and 24 different counterfeit banknotes (12 x £20 and 12 x £50 notes) were used as stimuli. Genuine notes were sourced from local commercial banks and showed typical signs of prior use. Counterfeit notes, obtained from the Bank of England, had been recovered from general circulation and showed a comparable level of wear. The primary obvious security features were a foil hologram stripe (£20) and green micro-optic security stripe (£50) as shown in see Fig. 6A. Counterfeit exemplars varied modestly from each other. Differences between counterfeit and genuine included general image resolution, contrast, and hue, as well as specific differences in the appearance of security features.

To prepare stimuli for presentation, each banknote was videoed in a well-lit room with natural light sources in front of the banknotes using a *Sony* FDR AX33BDI camcorder, recorded in 3840 × 2160 resolution (ultra high definition). During filming, each banknote was centred in a note frame attached to a robotic arm that rotated slowly around a vertical axis. This apparatus was positioned in front of a monochrome textured board. Each video was then edited into a 300 ms clip for use in the study. For £20’s, video clips were edited to include the security feature hologram’s image to visibly change between “£” and the number “20” and for a bright reflection from the shiny strip to be visible. For £50’s, editing included visible up-down motion in the holographic stripe and no obstruction by light glare. Half of the videos showed a note rotating left-to-right; remaining videos showed a note rotating in the opposite direction. Clips were then re-sized to match the physical size of each banknote. During testing, each clip was presented in the centre of the black laptop LCD screen framed by a grey (RGB: 127 127 127) rectangle. The participant viewed the screen from approximately 50 cm.

### Procedure

Participants were handed a genuine (physical) £20 then £50 banknote (order counterbalanced across participants) and asked to familiarise themselves with each note for one minute in preparation for the upcoming authentication task. Next, they viewed a genuine £20 and £50 banknote video clip on the screen for 3 s, appearing as it would be presented more briefly in the upcoming authentication task. These presentations were repeated at least five times and up to 10 times if requested, allowing participants to became familiar with the appearance of genuine notes. After familiarisation, the authentication task was conducted. On each trial, the participant viewed a bright, grey rectangle for a jittered interval (average 2 s), followed by a 300 ms banknote video clip. See Fig. 6B. At offset, the participant reported whether the banknote was real or fake by pressing the ‘z’ or ‘m’ key with their left and right index finger, respectively. The next trial began immediately after the response.

The authentication task comprised 20 blocks of 36 trials each (totalling 720 trials). Half of blocks presented only £20 s, remaining blocks presented only £50 s. Blocks alternated denomination with order being counterbalanced across participants. 20% of trials within each block presented counterfeit; order of banknote type within each block was individually pseudo-randomised. Before starting the experimental trials, six practice trials were provided using the same procedure as in the main experimental blocks except that feedback (beep for correct responses) was provided for practice trials only. The number of correct and incorrect trials for each banknote authenticity (genuine, counterfeit) used in the behavioural and EEG analyses are provided in Table 1. The number of trials, participants, and effects size indicate that the study was sufficiently powered (> 0.80)^39^.

**Table 1:** Number of correct and incorrect trials for each banknote authenticity type used in the behavioural and EEG analyses.

Banknote type	Number of incorrect trials		Number of correct trials	
	Genuine	Counterfeit	Genuine	Counterfeit
Behavioural	185.8	47.0	414.1	73.0
	*19.6*	*5.3*	*19.6*	*5.3*
EEG	198.6	54.2	401.3	65.8
	*20.4*	*5.4*	*20.4*	*5.4*

S.e in italics.

#### EEG recording

Electroencephalographic (EEG) data was collected during the authentication test. The EEG was recoded using active Ag–AgCl electrodes (BioSemi) from 32 scalp sites (FP1, FP2, AF3, AF4, F7, F8, F3, F4, FC5, FC6, FC1, FC2, T7, T8, CP5, CP6, CP1, CP2, C3, C4, P7, P8, P3, P4, PO3, PO4, O1, O2, Fz, Cz, Pz, Oz) and the left and right mastoids according to the 10–20 system (American Electroencephalographic Society, 1994). For the detection of eye movements and blinks, vertical and horizontal electro-oculogram (EOG) was recorded from electrodes placed above and below the right eye and at the outer canthi of each eye. The EEG and EOG were low-pass filtered with a fifth-order sinc filter (half-power cutoff at 128 Hz) and digitized at 512 Hz.

#### ERP processing

All ERP data analysis was conducted using EEGLAB^40^ and ERPLAB Matlab toolboxes^41^, as follows. The EEG signals were offline referenced to the average of the left and right mastoids. The EEG was bandpass filtered offline using a Butterworth infinite impulse response filter with half-power cutoffs at 0.05 and 30 Hz and a roll-off of 12 dB/octave. The data was then down-sampled to 256 Hz.

Noisy channels were substituted by interpolating neighbouring electrode sites. Then, independent component analysis was used to estimate and remove eye-blinks and eye-movements from the stimulus presentation period of the trial using EyeCatch^42^. Finally, trials were removed if the EEG exceeded ± 100 µV in any channel between 200 ms before and 1200 ms post banknote onset: these included noisy segments (eye-blinks, movement, muscle tensing, etc.…). Trials were also excluded if the vertical EOG exceeded ± 80 µV between 200 ms prior to and 200 ms post banknote onset to ensured that the eyes were not closed when the stimuli were presented.

Averaged event-related potential (ERP) waveforms were computed by averaging trials (200 ms before the onset of the banknote to 1200 ms post onset), after they were baseline corrected to the pre-stimulus interval. The amplitude of the P3 and extended P3 components were measured on the Pz electrode site, as the mean voltage during predefined time windows. The time windows for the P3 and extended P3 components were 450–600 ms and 600–900 ms post banknote onset, respectively. The P1 was defined as the mean voltage from 50 to 100 ms post banknote onset on the Oz channel.

#### Multivariate pattern analysis

Pre-processing of the EEG data for the MVPA analysis was the same as for the ERP analysis. MVPA was performed on the epoched data (200 ms prior to banknote onset to 1200 ms post onset) using the ADAM toolbox^28^ in Matlab. A linear discriminant classifier was trained and tested on each time point using a fivefold cross validation. The area under the curve (AUC) was used to measure classification accuracy. The classifier was first trained on the authenticity of the banknote (genuine versus counterfeit). The classifier was then trained separately for genuine and counterfeit banknotes depending on the response of the participant (genuine versus counterfeit response). Because of unbalanced trial numbers for genuine and counterfeit banknotes, the trial numbers were balanced based on the condition with the lowest trial numbers. Next, generalisation matrices were calculated using cross-classification across time. Statistical analyses for the MVPA were performed using the ADAM toolbox. Cluster-based permutation corrected 2-sided *t*-tests against chance (0.5) were used to analyse the classification accuracy.

#### Data analysis

Three 2 × 2 repeated-measures analyses of variance (ANOVAs) were conducted on ERP magnitude measures using denomination (£20, £50) and authenticity (Genuine, Counterfeit) as within-subject factors. The first ANOVA was conducted on the P1 (50–100 ms) component data at electrode Oz, the second on the P3 (450–600 ms) component data and the third on the extended P3 (600–900 ms) component data, both at electrode Pz.

To analyse the difference between conscious and unconscious neural activity to the banknotes, three further 2 × 2 repeated-measures ANOVAs were conducted on the same ERP components using authenticity (Genuine, Counterfeit) and response (Real, Fake) as within-subject factors. The data was collapsed across denomination to increase the number of correct and incorrect trials in the analysis. From these data, only participants who had more than 20 incorrect responses to both genuine and counterfeit notes were included (19 participants).

Counterfeit sensitivity was calculated as d′ for each authentication denomination. d′ was calculated as *Z* (hit rate)—*Z* (false alarm rate). Hit rate was calculated as the proportion of counterfeit notes recognised as counterfeit; False alarm rate was calculated as the proportion of genuine notes incorrectly judged as counterfeit. d′ was analysed using a paired-samples *t*-test comparing denominations (£20, £50). Reaction times (RT) faster than 200 ms, slower than 5000 ms, and incorrect trials were removed, then RT slower than 3 S.D above the participant x condition mean RT were removed. Remaining RT were analysed using a 2 × 2 repeated-measures ANOVA with denomination (£20 versus £50) and authenticity (genuine, counterfeit) as within-subject factors was conducted.

All follow-up pairwise comparisons were corrected for multiple comparisons using the False Discovery Rate procedure^43^. Alpha levels were set at 0.05 throughout.

### Ethical approval

All methods were carried out in accordance with relevant guidelines and regulations of the Research Ethics Committee at the University of Birmingham.
